# Fowlpoxvirus recombinants coding for the *CIITA* gene increase the expression of endogenous MHC-II and Fowlpox *Gag/Pro* and *Env* SIV transgenes

**DOI:** 10.1371/journal.pone.0190869

**Published:** 2018-01-31

**Authors:** Massimiliano Bissa, Greta Forlani, Carlo Zanotto, Giovanna Tosi, Carlo De Giuli Morghen, Roberto S. Accolla, Antonia Radaelli

**Affiliations:** 1 Department of Pharmacological and Biomolecular Sciences, University of Milan, via Balzaretti 9, Milan, Italy; 2 Department of Experimental Medicine, University of Insubria, Via O. Rossi 9, Varese, Italy; 3 Department of Medical Biotechnologies and Translational Medicine, University of Milan, via Vanvitelli 32, Milan, Italy; 4 Catholic University “Our Lady of Good Counsel”, Rr. Dritan Hoxha, Tirana, Albania; 5 CNR Institute of Neurosciences, Cellular and Molecular Pharmacology Section, University of Milan, via Vanvitelli 32, Milan, Italy; Uniformed Services University, UNITED STATES

## Abstract

A complete eradication of an HIV infection has never been achieved by vaccination and the search for new immunogens that can induce long-lasting protective responses is ongoing. Avipoxvirus recombinants are host-restricted for replication to avian species and they do not have the undesired side effects induced by vaccinia recombinants. In particular, Fowlpox (FP) recombinants can express transgenes over long periods and can induce protective immunity in mammals, mainly due to CD4-dependent CD8^+^ T cells. In this context, the class II transactivator (CIITA) has a pivotal role in triggering the adaptive immune response through induction of the expression of class-II major histocompatibility complex molecule (MHC-II), that can present antigens to CD4^+^ T helper cells. Here, we report on construction of novel FP*gp* and FP*env* recombinants that express the highly immunogenic SIV Gag-pro and Env structural antigens. Several FP-based recombinants, with single or dual genes, were also developed that express CIITA, driven from H6 or SP promoters. These recombinants were used to infect CEF and Vero cells *in vitro* and determine transgene expression, which was evaluated by real-time PCR and Western blotting. Subcellular localisation of the different proteins was evaluated by confocal microscopy, whereas HLA-DR or MHC-II expression was measured by flow cytometry. Fowlpox recombinants were also used to infect syngeneic T/SA tumour cells, then injected into Balb/c mice to elicit MHC-II immune response and define the presentation of the SIV transgene products in the presence or absence of FP*CIITA*. Antibodies to Env were measured by ELISA. Our data show that the H6 promoter was more efficient than SP to drive *CIITA* expression and that *CIITA* can enhance the levels of the *gag/pro* and *env* gene products only when infection is performed by FP single recombinants. Also, CIITA expression is higher when carried by FP single recombinants than when combined with FP*gp* or FP*env* constructs and can induce HLA-DR cell surface expression. However, *in-vivo* experiments did not show any significant increase in the humoral response. As CIITA already proved to elicit immunogenicity by improving antigen presentation, further *in-vivo* experiments should be performed to increase the immune responses. The use of *prime/boost* immunisation protocols and the oral administration route of the recombinants may enhance the immunogenicity of Env peptides presented by MHC-II and provide CD4^+^ T-cell stimulation.

## Introduction

The Human Immunodeficiency Virus (HIV) is the aetiological agent of the acquired immunodeficiency syndrome pandemic, a sexually transmitted disease for which many drugs have been developed for both single and combined therapies. These pharmacological treatments have led to a chronic trend of the disease and to longer survival. With the exception of the “Berlin patient” [1], where AIDS was cured by bone marrow transplantation, complete eradication of infection has never been achieved. Despite relatively positive results compared to previous trials, the RV144 Thai vaccine trial demonstrated only modest and transient protection against HIV-1 acquisition [2], and the search for new immunogens that can induce long-lasting protective responses is ongoing.

Live-attenuated viral vaccines are among the most effective immunogens against infectious diseases [3, 4], as they are potent stimulators of antibodies and CD8^+^ cytolytic T lymphocytes, and protect against both homologous and heterologous virus strains [5, 6]. However, the development of a live-attenuated HIV vaccine is precluded by the risk of the emergence of virulent revertants [7].

As DNA vaccines are weakly immunogenic in primates when used alone, and live viral vaccine recombinants are sometimes less effective due to the immune response to the vector [8], these two approaches have often been combined in *prime-boost* vaccination strategies [9, 10]. In this context, avipox viruses have taken on an important role in the development of novel recombinant immunogens, as they are host-restricted for replication to avian species, although permissive for entry and transgene expression in most mammalian cells [9, 11–13]. Moreover, avipoxvirus vectors do not cause the undesired side effects induced by vaccinia recombinants, and they are not neutralised in individuals who are already immunised against smallpox [14]. In particular, Fowlpox (FP) recombinants can express foreign antigens for long periods, to induce protective immunity in mammals [15–18]. FP recombinants can also elicit IFN-γ responses, mainly due to CD4-dependent CD8^+^ T cells, which are specific for HIV and chimeric Simian-Human Immunodeficiency Virus (SHIV) gene products [19–21].

Env-encoded glycoproteins are the only antigens of HIV and HIV-infected cells that are accessible to antibodies, and follow-up analyses of the RV144 Thai trial showed that the humoral response to the V1/V2 regions of the Env protein is associated with reduced risk of HIV-1 acquisition [2, 22, 23]. Studies on rhesus monkeys have also demonstrated partial protection by adenovirus and avipoxvirus recombinants against Simian Immunodeficiency Virus (SIV) [24], and an association of Env-specific antibodies with decreased risk of infection [25]. However, multiple evasion mechanisms have been developed by HIV to escape the host humoral immune response, such as a flexible conformation [26], highly variable loops [27], and carbohydrate moieties that can shield potentially conserved epitopes, thus limiting the elicitation of broadly neutralising antibodies [28]. Despite of the recent progress in the identification of such broadly neutralising antibodies [29–32], the development of an effective vaccine that protects against the majority of HIV strains is still a challenge and might depend on the possibility of translating cross-specific antigens into powerful immunogens that can induce protective responses.

Several studies have also demonstrated that co-expression of the *gag*, *pol* and *env* genes resulted in improved containment of disease progression than the expression of the *env* gene alone [33, 34]. The SIV_mac_ *gag* gene product, which is antigenically cross-reactive with HIV-1 *gag* [35], has already been shown to be important in the induction of protective immunity, and Gag-specific cellular immune responses appear to be important in the control of viremia in HIV-infected humans [36–38] and SIV-infected monkeys [24, 39, 40].

The ability to induce protective antigen-specific immune responses is one of the major problems in the preparation of an HIV vaccine. T-cell immunity is needed to control pre-existing virus infections, and CD4^+^ T helper (Th) cells are necessary for long-lasting CD8^+^ cytolytic T lymphocytes, as well as for prophylactic humoral responses and B-cell memory.

Although humoral immunity has little effect on the progress of established HIV-1 infections [41], non-neutralising antibodies can trigger antibody-dependent cellular cytotoxicity [42] and help to control established infections [43], as was also shown in the follow-up analysis of the RV144 Thai trial [44]. Neutralising antibodies can apparently prevent infections [29], although the preparation of the native Env trimers that are required to elicit neutralising antibodies is still a challenge [45] because the native trimeric folding is not stable. There are also additional problems related to the development of neutralising antibodies in only a small percentage of HIV-1–infected individuals (10%-30%) and only at the later stages of natural infections [46, 47]. These findings have shown that humoral immunity alone is not sufficient to promote an effective immune response against HIV-1 infection, and they suggest that the activation of CD4^+^ Th cells, which is fundamental for optimal induction of both humoral and cellular effector mechanisms, might represent the strategy to limit HIV-1 infection.

The *AIR-1*-encoded class II transactivator (CIITA) is the master regulator of the class-II major histocompatibility complex (MHC-II) genes [48, 49]. Through induction of expression of MHC-II molecules, which present antigens to CD4^+^ Th cells, CIITA has a pivotal role in triggering adaptive immune responses [50]. Ectopic expression of CIITA in MHC-II–negative tumour cells can render these cells MHC-II–positive. This endows them with the ability to present their own tumour antigens to CD4^+^ Th cells *in vivo* and to trigger potent and protective anti-tumour adaptive immune responses in both the CD4^+^ and CD8^+^ T-cell compartments [51, 52]. CIITA has also been shown to have antiretroviral properties, by acting as a host restriction factor against human oncogenic retroviruses [53–56], and also against HIV. For HIV, CIITA was shown to inhibit viral replication in both T cells and monocytes through suppression of the Tat transactivating function on the viral long terminal repeat [57, 58]. Thus, CIITA has a dual action against retroviruses: it promotes the effector mechanisms of the adaptive immunity, and it controls viral replication through its intrinsic antiviral activity.

The final goal of the present study was to combine within the same cell the capacity to express SIV viral antigens and CIITA-induced MHC-II molecules, which can be instrumental to the optimal presentation of viral antigens to CD4^+^ T cells.

Here, we report on the construction of different FP recombinants that express CIITA either alone or in combination with the highly immunogenic Env and Gag-Pro structural viral antigens. These novel FP constructs have been used for infection or co-infection of CEF and Vero cells *in vitro* to determine transgene expression. The recombinants were also used for infection of murine T/SA mammary carcinoma cells [59, 60] to assess *in vivo* the induction of MHC-II expression and the possible presentation of MHC-II-bound viral transgene products, which may result in the increase of the immune response. Transcript and protein expression were verified by real-time PCR, Western blotting (WB), and flow cytometry. The subcellular localisation of the different proteins was also assessed by confocal microscopy.

Co-infection experiments using FP recombinants expressing Gag/Pro or Env with CIITA demonstrated that CIITA can increase the levels of *gag/pro* and *env* gene products. This occurs only when the *CIITA* gene is driven by the H6 promoter and infection is performed with FP single recombinants. This suggests the lower efficiency of the synthetic SP Vaccinia virus promoter as well as the uselessness of constructing double recombinants, the preparation of which is often very troublesome. Although *in-vivo* experiments using Balb/c mice injected with syngeneic T/SA tumour cells, infected with FP recombinants, did not show any significant increase of the immune response to viral antigens, as assessed by the very low antibody levels in the serum, the complete cellular immune responses will be the object of further investigations.

## Materials and methods

### Cells

Specific-pathogen-free primary chick embryo fibroblasts (CEFs) were grown in Dulbecco’s Modified Eagle’s Medium (DMEM) supplemented with 5% heat-inactivated calf serum (CS, Gibco Life Technologies, Grand Island, NY, USA), 5% Tryptose Phosphate Broth (Difco Laboratories, Detroit, MI, USA), 100 U/mL penicillin, and 100 μg/mL streptomycin (P/S). Green monkey kidney (Vero) cells and normal human lung fibroblasts (MRC-5) were grown in DMEM supplemented with 10% heat-inactivated CS with P/S. Human embryonic kidney 293T cells (kindly provided by B.M. Peterlin, UCSF, San Francisco, USA), and Balb/c-mice-syngeneic T/SA mammary tumour cells (kindly provided by S. Ferrini, IST Genova, Italy) were grown in DMEM with 10% heat-inactivated foetal calf serum (FCS, Gibco Life Technologies). Replication competent CEFs were used to grow and titrate the recombinants, but also to evaluate protein expression in WB analysis. Replication non-permissive Vero cells were also used in WB analyses as a prototype of non-human primate mammalian cells and to prepare the plate-bound antigen used in ELISA after lysing cells infected with the FP*envM766* recombinant. 293T cells were used as prototype of human cells to compare transgene expression by the single and double recombinants when driven by different promoters. These cells were also used to evaluate the expression of CIITA-driven MHC-II expression (HLA-DR in humans) by immunofluorescence and flow cytometry or confocal microscopy after infection with the different recombinants. T/SA mammary cells, infected with different recombinants, were used as a prototype of mouse tumour cells, to assess the expression of mouse MHC-II molecules after CIITA expression by the recombinants. These cells were inoculated in syngeneic Balb/c mice to determine the humoral immune responses against viral products assuming that these cells can present these products via the induced CIITA-mediated MHC-II molecules.

### Recombination plasmids

Different plasmids were prepared for *in-vitro* recombination, which contained the SIVmacM766 *gag/pro* or the *env* genes, either alone or with the *CIITA* gene. The SIV *gag/proM766* gene was isolated from a macaque infected with the M766 variant of SIVmac251 [61, 62], and it includes the complete *gag* and protease sequences to which two transcription termination codons have been added. The *envM766* gene includes the whole *env* sequence except for a 5’ 19-nt fragment, which was substituted by a tPA membrane translocation signal that was removed during protein maturation. Both the *gag/proM766* and *envM766* genes (here referred to as *gp* and *env*, respectively) were codon optimised for expression in humans, except for the regulatory sequences, as they were prepared for human vaccination as an ultimate goal.

The *gp* and *env* genes were obtained from the pCM766*gag/pro* and pCM766*env* plasmids (kind gift from G. Franchini, National Cancer Institute, NIH, Bethesda, MD, USA). In particular, the *gp* gene was excised using HindIII/ SphI restriction enzymes (Fermentas UAB, Vilnius, Lithuania), subcloned into pUC19 and inserted into pFP_MCS_ using HindIII/ SalI. Non-coding sequences upstream and downstream of the *gp* gene and unnecessary sequences (i.e., GFP gene) were removed enzymatically [63]. The *env* gene was excised using the HindIII/ KpnI enzymes, inserted into pFP_MCS_ and the upstream non-coding sequence was deleted as well. The SIV *gp* and *env* genes were thus inserted downstream of the Vaccinia virus H6 (H6) early/ late promoter [64], and inside the 3-β-hydroxysteroid dehydrogenase 5-delta 4 isomerase gene. The correct insertion was verified by PCR, restriction analyses, and by sequencing the ligation ends of the recombinant plasmid. The *CIITA* gene was amplified from pcFlag*CIITA* [53] using the primers V355 (forward; 5’ GAG AAG ATC TAT GCG TTG CCT GGC TCC ACG 3’) and V356 (reverse; 5’ TTC CCC CGG GGT CGA CAT AAA AAT CAT CTC AGG CTG ATC CG 3’). For cloning, primers were added with the BglII and SalI-SmaI restriction sites at the 5’ and 3’ ends of the gene, respectively. The T5NT sequence was also added, as an additional poxviral transcription termination signal. Reactions were performed with 3 mM MgCl_2_, 200 μM dNTPs, 1 μM primers, and 0.025 U/μL Expand High Fidelity DNA polymerase (Roche Applied Science, Mannheim, Germany). After denaturation at 94°C for 1 min, the amplification was performed for 25 cycles at 94°C for 30 s, 60°C for 30 s, and 72°C for 3 min, followed by 72°C for 7 min. The whole gene was sequenced before cloning, to exclude any possible mistake due to the PCR amplification.

The pFP*CIITA*_*SP*_ plasmid was constructed by cloning (downstream of the SP synthetic promoter [65]) the human *CIITA* gene (cut with BglII/ SalI) into pFP_MCS-GFP_ (cut with BamHI/ SalI), through the compatible ends of BglII and BamHI. The pFP*gpCIITA* and pFP*envCIITA* double-recombination plasmids were obtained by transferring the *CIITA*_SP_ sequence into pFP*gp* and pFP*env*, respectively, in the reverse sense to the H6 promoter. In particular, two *CIITA* fragments were obtained from pFP*CIITA*_*SP*_ using SalI/ KpnI. The first was subcloned into pBluescriptII (Stratagene, La Jolla, CA, USA), and then transferred downstream of the *gp* (or *env*) gene of pFP*gp* (or pFP*env*) after digestion with NotI/ KpnI. The second was cloned downstream of the first fragment in the KpnI site. The correct orientation of the second fragment was verified by restriction analysis, and the integrity of the whole *CIITA* gene was verified by sequencing. Plasmid pFP*CIITA*_H6_ was obtained after excision with SalI/ HincII of the *CIITA* gene, which had previously been subcloned into pTOTO_1101_ (kind gift from C. Rice, The Rockefeller University, NY, USA). *CIITA* was then inserted downstream of the H6 promoter [64, 66] of pFP_MCS_, digested with SalI/ SmaI.

### Recombinant fowlpox viruses

The FP*gpCIITA* and FP*envCIITA* double recombinants, and the FP*gp*, FP*env*, FP*CIITA*_H6_ and FP*CIITA*_SP_ single recombinants were generated by *in-vivo* double crossing-over homologous recombination [63, 66], using the plasmids described above. For clarity, the promoters of the transgenes inserted into the FP recombinants are listed in Table 1. The recombinants were selected by their transgene expression, amplified in CEFs, purified on discontinuous sucrose density gradients, and titrated essentially as described previously [67]. Briefly, the cells were harvested, ultracentrifuged at 30,000× *g* for 2 h at 4°C, and the pellets were resuspended in 1 mM Tris, 150 mM NaCl, 1 mM EDTA, pH 7.4. After adding 0.06% trypsin, the pellet was incubated for 5 min at 37°C, and the virus was released from the cells by sonication. The supernatant was overlaid onto a discontinuous 30% to 45% (w/w) sucrose gradient, in the same buffer. After ultracentrifugation at 38,000× *g* for 1 h, the viral band at the interface was recovered, diluted with 1 mM Tris-HCl, pH 9, and pelleted at 67,000× *g* for 1 h. The purified virus was resuspended in Ca^2+^-free and Mg^2+^-free phosphate-buffered saline (PBS^-^), sonicated, aliquoted, and frozen at -80°C until use.

**Table 1:** Promoters inserted into the double or single FP recombinants for the expression of the *gp*, *env*, or *CIITA* transgenes.

double recombinants		promoter	
	*gp*	*env*	*CIITA*
FP*gpCIITA*	H6		SP
FP*envCIITA*		H6	SP
**single recombinants**			
FP*gp*	H6		
Fp*env*		H6	
FP*CIITA*_*SP*_			SP
FP*CIITA*_*H6*_			H6

H6: vaccinia virus early/ late promoter

SP: synthetic promoter

### Real-time PCR

Total RNA was extracted from Vero cells infected with 0.5 PFU/cell of each of the FP*gp*, FP*env*, FP*CIITA*_H6_, FP*CIITA*_SP_ single recombinants, and the FP*gpCIITA* and FP*envCIITA* double recombinants, or co-infected with the FP*gp*+FP*CIITA*_H6_ or FP*env*+FP*CIITA*_H6_ single recombinants. Harvesting was performed on days 1, 3, and 6 post-infection (p.i.), as described previously, with minor modifications [68]. Briefly, after lysis, the RNAs were extracted using the TRIzol LS reagent (Invitrogen), according to the manufacturer instructions. Reverse transcriptions were performed in a final volume of 50 μL, using 3 μg RNA and High Capacity cDNA Archive kits (PE Applied Biosystems, Foster City, CA, USA). The reactions were run at 25°C for 10 min, followed by 37°C for 2 h, and 4°C. The cDNA (5 μL) was then added to each well of a MicroAmp Optical 96-well reaction plate (Applera, PE Applied Biosystems) in the presence of 2× Power SYBR green master mix, using the primers V406 (forward; 5’ ACC ACG TGA TGG CCA AAT G 3’) and V407 (reverse; 5’ CTT CTT TCC CCA TGG ACC AA 3’) to identify the *gp* gene, V408 (5’ GCA CCC GGA TGA TGG AAA C 3’) and V409 (5’ V409 GTA GTA CTT GTT CAG GCT GAT GAT GGT 3’) for the *env* gene, V380 (5’ TGG AGA TGA ACT CGG ACC TC 3’) and V381 (5’ CGA CCA CCA CCA ACT TCA A 3’) for the housekeeping RPS7 human gene, and V414 (5’ TCT TAA CAG CGA TGC TGA CCC 3’) and V415 (5’ TCG CAG TTG ATG GTG TCT GTG 3’) for the *CIITA* gene, in a final volume of 25 μL. FPwt-infected Vero cells were used as negative controls, while Vero cells transfected for 24 h with the pcFlag*CIITA*, pCM766*gp* and pCM766*Env* plasmids were used as positive controls. All of the reactions were performed in an ABI PRISM 7700 apparatus (PE Applied Biosystems). The PCR conditions were 50°C for 2 min, 95°C for 15 min, followed by 40 cycles at 95°C for 15 s and 60°C for 1 min. A 20-min dissociation protocol was also applied. Two different analyses were performed and the copy numbers of the *gp*, *env* and *CIITA* transcripts were calculated using the comparative C_t_ method (also known as the 2^-[delta] [delta]Ct^ method), where [delta]C_t, sample_ = C_t, gp/env/CIITA_—C_t, RPS7_, and [delta]C_t, sample_ is the C_t_ value for any sample normalised to the RPS7 endogenous housekeeping transcripts and [delta] [delta]C_t_ = [delta]_Ct, sample_—[delta]C_t, wt_. In all of the samples, 2^-[delta] [delta]Ct^ refers to an N-fold increase in the *gp/env/CIITA* copy numbers relative to the FPwt control.

### Western blotting

To determine whether the *gp*, *env* and *CIITA* proteins were expressed, CEFs and Vero cells were infected using the single and double recombinants (10 PFU/cell) and examined by WB, after loading the same amount of proteins, as described previously [69, 70]. The experiments were repeated twice with similar results. Co-infection with single recombinants was also performed with Vero cells using 8 PFU/cell/each recombinant. The blotted nitrocellulose membranes were incubated overnight at 4°C using specific antibodies. For *gp* and *env*, anti-SIV monkey polyclonal antibodies were used (1:100-dilution; kind gift from J. Heeney, Department of Veterinary Medicine, University of Cambridge, UK), followed by rabbit anti-monkey horseradish-peroxidase (HRP)-conjugated serum (1:2,000 dilution; Sigma, St Louis, MO, USA). The anti-CIITA mouse monoclonal antibody (mAb) (1:500-dilution; 7-1H; Santa Cruz Biotechnology, Santa Cruz, CA, USA) was used, followed by goat anti-mouse HRP-conjugated serum (1:1,000 dilution; DakoCytomation, Carpinteria, CA, USA). After a 1-h incubation and 2-h washes, the proteins were revealed using the ECL system (EuroClone, Pero, Milan, Italy). Cells infected with FPwt were used as the negative control.

### Flow cytometry analysis

HLA-DR and MHC-II cell surface expression was assessed by immunofluorescence and flow cytometry (BD FACS aria II, Beckman Coulter, Milan, Italy). Briefly, human 293T or mouse T/SA cells (6 ×10^5^) were infected or co-infected with 4, 6 or 8 PFU/cell with single or double recombinants that expressed *CIITA* driven by either the H6 or the SP promoter and the SIV *gp* or *env* genes, always driven by the H6 promoter. After infection, cells were washed twice with PBS^-^, dissociated with trypsin-EDTA, and resuspended in complete medium (DMEM, 10% FCS, P/S). Cells (3 ×10^5^/tube) were pelletted at 800× *g* for 5 min, washed, the supernatant was discarded and the pellet resuspended in PBS^-^ for FACS analysis. In particular, for the *env* gene, 293T cells were infected with: FP*env* (8 PFU/cell), FP*envCIITA* (8 PFU/cell), FP*CIITA*_*SP*_ (8 PFU/cell), FP*CIITA*_H6_ (8 PFU/cell), FP*env*+FP*CIITA*_H6_ (4+4 PFU/cell), or FP*env*+FP*CIITA*_H6_ (8+8 PFU/cell). For the *gp* gene, the corresponding *gp* single recombinants were used for 293T cells, i.e.: FP*gp* (8 PFU/cell), FP*gp*+FP*CIITA*_H6_ (4+4 PFU/cell), or FP*gp*+FP*CIITA*_H6_ (8+8 PFU/cell). The 293T cells were transiently transfected with 1 μg of the pcFlag*CIITA* expression vector [71] and used as a positive control of HLA-DR expression. For the *env* gene, mouse T/SA cells were infected with: FP*env* (8 PFU/cell), FP*CIITA*_H6_ (8 PFU/cell), FP*env*+FP*CIITA*_H6_ (4+4 PFU/cell), or FP*env*+FP*CIITA*_H6_ (8+8 PFU/cell). For the *gp* gene, T/SA cells were infected with: FP*gp* (8 PFU/cell), FP*gp*+FP*CIITA*_H6_ (4+4 PFU/cell), or FP*gp*+FP*CIITA*_H6_ (8+8 PFU/cell). The following monoclonal antibodies were used as primary antibodies: MKD6 (rat anti-mouse MHC-II I-A/I-E mAb, undiluted) or D1-12 (mouse anti-human MHC-II HLA-DR, undiluted). As secondary antibodies, FITC-conjugated goat anti rat and anti mouse F(ab)2 mAb (Sigma) were used. All antibodies were incubated for 30 min at 4°C.

### Confocal microscopy analysis

Human 293T cells (2.5 ×10^5^) were seeded onto glass coverslips in 24-well plates, and infected with FP*CIITA*_*SP*_ (8 PFU/cell), or FP*CIITA*_H6_ (8 PFU/cell), or FP*env* (8 PFU/cell) recombinants, or co-infected with FP*env*+FP*CIITA*_H6_ (8+8 PFU/cell), to determine *CIITA* subcellular localisation and expression of the cell-surface MHC-II HLA-DR. For the *gp* gene, FP*gp* (8 PFU/cell) or FP*gp*+FP*CIITA*_H6_ (both 4+4 and 8+8 PFU/cell) were used. After 48 h, the cells were fixed with cold methanol, washed three times with PBS^-^, and blocked for 1 h with PBS^-^ containing 0.5% bovine serum albumin (Sigma), as described previously [72]. The cells were incubated for 30 min at 4°C either with an anti-SIV monkey polyclonal serum (1:200 dilution; kind gift from C. Stahl-Hennig, German Primate Centre, Goettingen, Germany), or with an anti-human MHC-II HLA-DR mAb (undiluted; clone D1.12) [73], or with an anti-*CIITA* mouse mAb (1:200-dilution; anti-*CIITA* 7-1H; Santa Cruz Biotechnology) The cells were then washed five times with PBS^-^ before adding the secondary antibodies. To detect *gp-env*, FITC goat anti-monkey antibody (IgG/IgA/IgM, Sigma, 1:40 dilution; yellow) or FITC goat anti-human antiserum, also cross-reacting with monkey primary serum (Cappel MP Biomedicals, Aurora, OH, USA) were utilised. To detect HLA-DR, goat anti-mouse IgG2a Alexa Fluor 546 (1:200 dilution; violet) was used whereas IgG1 Alexa Fluor 633 (red) was used to detect CIITA. Secondary antibodies were all used for 30 min at 4°C in the dark.

All of the antibody dilutions were performed in 0.1% bovine serum albumin in PBS. After five washes, the samples were mounted with Fluor Save reagent (Calbiochem) and analysed under confocal microscopy (Leica TCS SP5) with a 63× objective lens, with wavelengths of 438 nm, 543 nm and 633 nm to detect the *env* or *gp* (yellow), HLA-DR (violet), or *CIITA* (red) proteins, before importing into the LAS AF software.

### *In-vivo* experiments

T/SA tumour cells, syngeneic with Balb/c mice (Charles River Laboratories, Como, Italy), were infected with different FP recombinants: FP*env* (4 PFU/cell), FP*CIITA*_H6_ (4 PFU/cell), and FP*env*+FP*CIITA*_H6_ (4+4 PFU/cell). MHC-II and Env expression by T/SA cells were verified 72 h later by FACS analysis. The cells were then treated with mytomycin C (50 μg/mL) for 1.5 h at 37°C and the absence of growth capacity was checked over time. The cells were inoculated subcutaneous (10^6^ cells in 200 μL RPMI/mouse) into four groups of 8-week-old female animals (5 mice/group). Group 1 received cells infected with FP*env*, Group 2 cells infected with FP*CIITA*_H6_, Group 3 cells infected with FP*env*+FP*CIITA*_H6_. Mock-infected cells were used for the control mice (Group 4). All of the animals were re-inoculated 3 weeks later. Blood was drawn before the first (T0) and second (T1) inoculations, and 2 weeks after the second inoculation (T2). Animals were handled and kept in strict accordance with the Guide for the Care and Use of Laboratory Animals, under the supervision of authorised personnel. The animals were monitored twice a week by visual inspection and palpation. All animal work was conducted according to relevant national and international guidelines and was approved by the University of Insubria Internal Ethical Committee CESA and by the Italian Ministry of Health.

### ELISA

The ELISA was essentially performed as described previously [74], using lysed Vero cells previously infected with 5 PFU FP*envM766* as the plate-bound antigen. Briefly, 96-well maxisorp microtitre plates (Nunc, Naperville, IL, USA) were coated with Vero lysates in PBS^-^ (5 ×10^4^ cells/well) in 0.05 M carbonate–bicarbonate buffer, pH 9.6, and incubated overnight at 4°C. The sera from all of the animals, drawn at T0 and at different times p.i. (T1, T2), were then added at 1:20 dilution, and the binding was revealed using goat anti-mouse HRP-conjugated serum (1:2000 dilution; Dako) and tetramethylbenzidine substrate (Sigma). The absorbance for each well was measured at 450 nm with a microplate reader (550; Bio-Rad Laboratories, Hercules, CA, USA).

### Statistical analyses

Statistical analyses were performed using one-way ANOVA parametric tests and Bonferroni analysis of variance, using the GraphPad Prism version 2.0 software (www.graphpad.com). Statistical significance was set as p <0.05 (*), p <0.01 (**), p <0.001 (***).

## Results

### CIITA is expressed at higher levels by the H6 promoter, and increases both *gp* and *env* transcripts in cells co-infected with the FP*gp*+FP*CIITA* or FP*env*+FP*CIITA* recombinants

**Fig 1:**
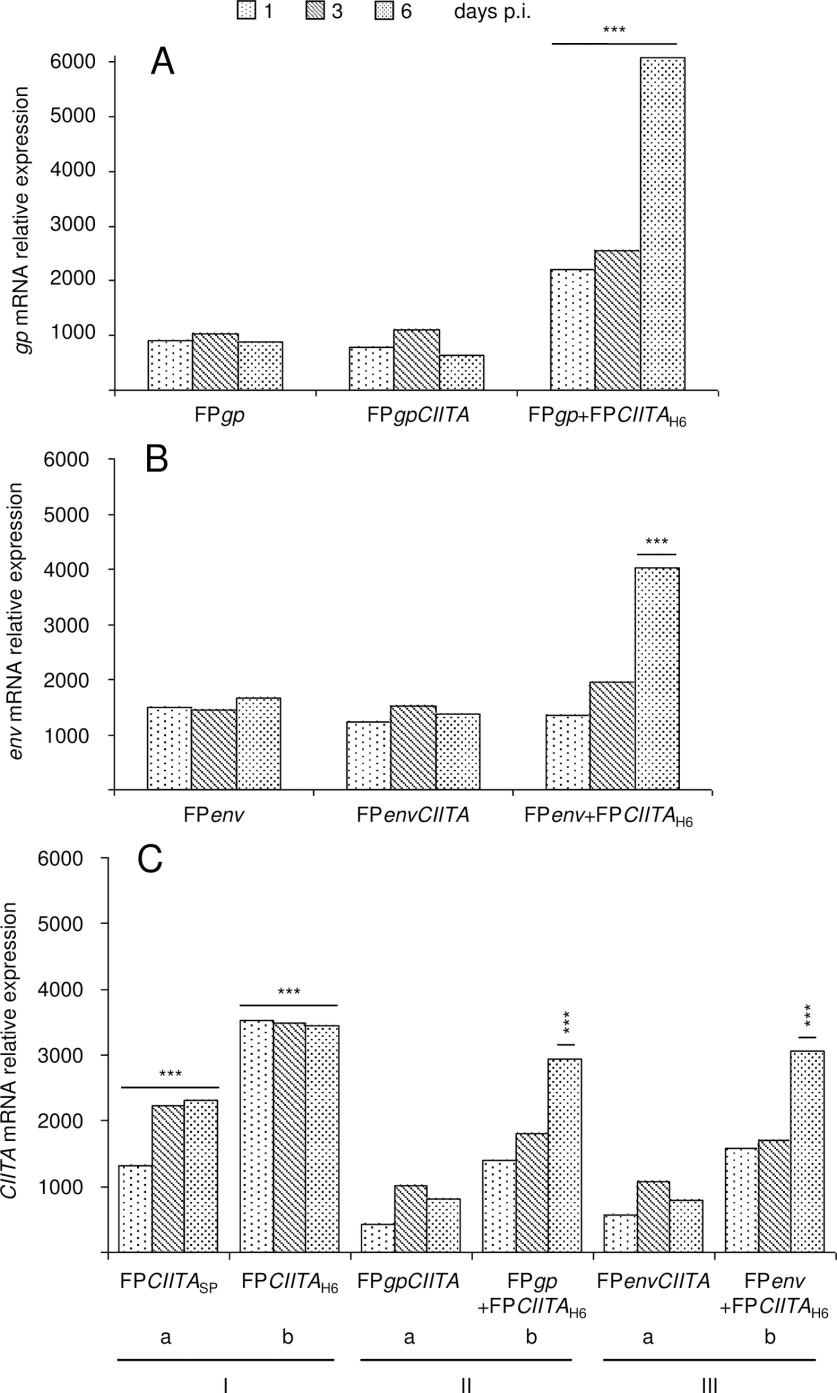
Time-course of the expression of the *gp*, *env* and *CIITA* transcripts in Vero cells using real-time PCR. After infection with the different recombinants, quantitative expression of the transgenes was evaluated at days 1, 3 and 6 p.i.. Co-infection with the FP*gp*+FP*CIITA*_H6_ or FP*env*+FP*CIITA*_H6_ recombinants significantly increased the *gp* and *env* expression compared to cells infected with either the FP*gp* single or FP*gpCIITA* double recombinants (A, B). Also, CIITA expression was significantly higher when the transgene was driven by the H6 promoter than by the SP promoter (C, I; p <0.001). *CIITA* expressed by FP*CIITA* alone either under the SP or H6 promoter is better expressed than when combined with the second *gp* or *env* transgene (panel C, Ia *vs* IIa and IIIa, and Ib *vs* IIb and IIIb, p <0.001). However, when co-infection with single recombinants is performed, its expression increases over time (panel C, IIb and IIIb, day 6 *vs* days 1 and 3, p <0.001). Mock-infected cells were always negative, as were cells infected with FPwt (data not shown). The experiment was repeated twice and the results are given as the average of the data. Data were normalised against the RPS7 endogenous housekeeping gene transcript, which indicates N-fold increases *vs* control mRNA at each time point. Statistical significances using one-way ANOVA parametric tests and Bonferroni analysis of variance are shown: p <0.01 (**), p <0.001 (***).

To determine the expression of the different transgenes, Vero cells were infected with the different FP recombinants, and the mRNA expression levels of *CIITA*, *gp* and *env* were analysed over time using real-time PCR (Fig 1). The experiment was repeated twice and the results are given as the average of the data. The expression levels of *gp* were constant up to day 6 p.i. (Fig 1A, FP*gp*), and was similar when co-expressed with *CIITA* by the FP*gpCIITA* recombinant (Fig 1A, FP*gp*CIITA). Interestingly, in cells co-infected with the FP*gp*+FP*CIITA*_H6_ recombinants, there were significant increases in *gp* mRNA levels at days 1, 3 and 6 p.i., as compared to cells infected with either the FP*gp* single transgene or the FP*gpCIITA* double transgene recombinants (p <0.001, FP*gp*+FP*CIITA*_H6_ *vs* FP*gp* and FP*gpCIITA*). Similar results were obtained using the *env* recombinants (Fig 1B), where the amounts of *env* mRNA were significantly increased in cells co-infected with the FP*env*+FP*CIITA*_H6_ recombinants (p <0.001), although only at 6 days p.i.. Thus, CIITA expressed by the FP*gpCIITA* and FP*envCIITA* double recombinants did not modify the amounts of *gp* or *env* mRNA compared to the FP*gp* or FP*env* single recombinants (Fig 1A and 1B). *CIITA* transcription was significantly greater when it was driven by the H6 promoter, rather than the SP promoter (Fig 1C, I; days 1, 3, 6, b *vs* a, p <0.001). Also, *CIITA* expressed by FP*CIITA* alone under the SP promoter is better expressed than by the double FP*gp*CIITA and FP*env*CIITA recombinants, where it is driven by the same SP promoter (panel C, Ia *vs* IIa and IIIa, p <0.001). Similarly, *CIITA* driven by the H6 promoter is better expressed when used alone than after co-infection (panel C, Ib *vs* IIb and IIIb, p <0.001), but its expression significantly increases over time (panel C, IIb and IIIb, day 6 *vs* days 1 and 3, p <0.001).

### In Vero cells, CIITA is better expressed under the control of H6 promoter, and co-infection of the FP*gp*+FP*CIITA*_H6_ or FP*env*+FP*CIITA*_H6_ recombinants increases Gp and Env protein expression

**Fig 2:**
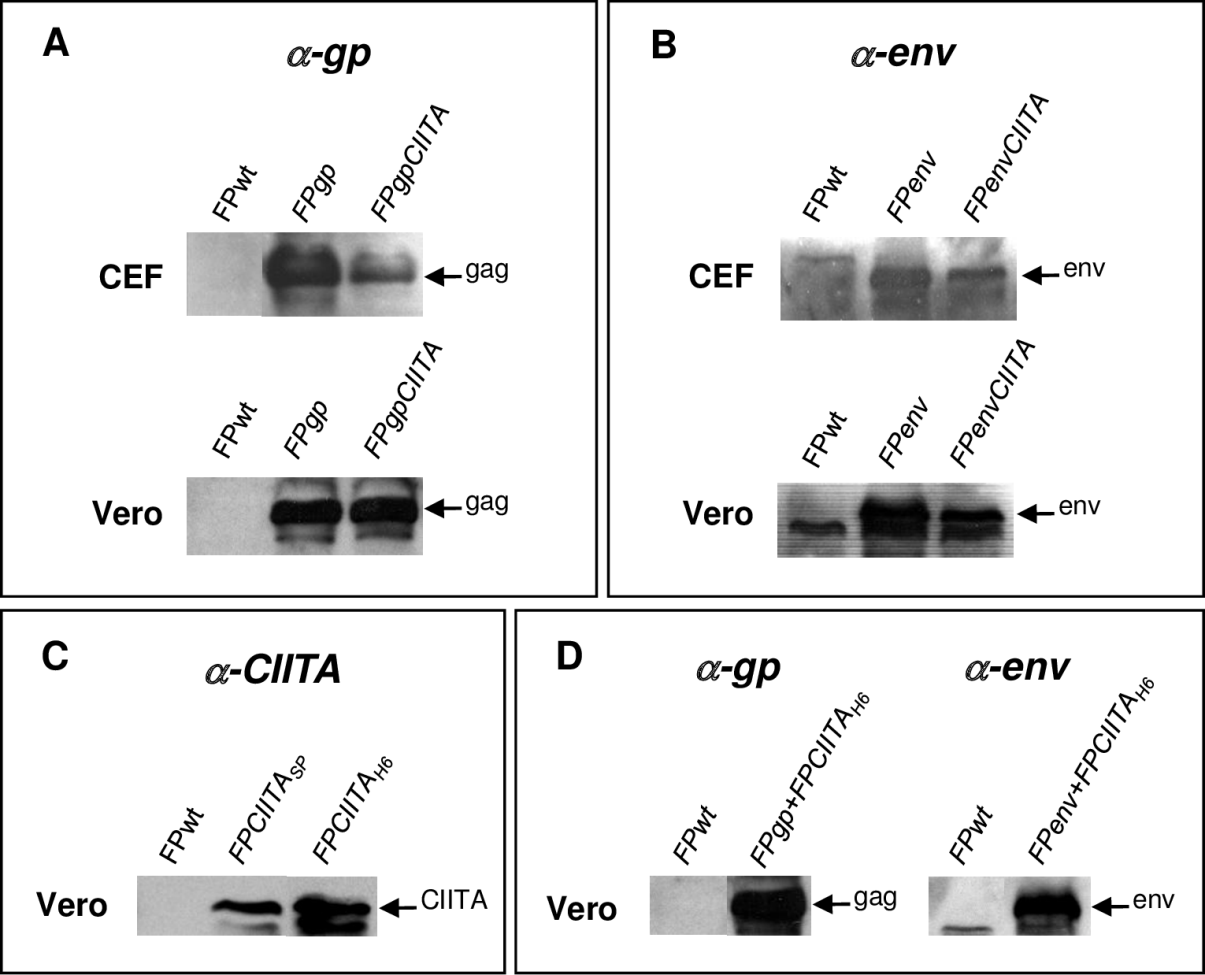
Expression of the Gp, Env and CIITA proteins by FP recombinants in CEFs and Vero cells. The cells were infected by the single or double recombinants and examined by WB to determine Gp (A), Env (B) and CIITA (C) protein expression. Gp and Env proteins were expressed following the infection of either CEFs or Vero cells with the FP*gpCIITA* or FP*envCIITA* double recombinants (A, B). The expression of CIITA was high when this was driven by the H6 promoter (C). Co-infection of Vero cells with either the FP*gp+*FP*CIITA*_H6_ or FP*env+*FP*CIITA*_H6_ recombinants showed high *gp* and *env* expression (D). The Gp and Env proteins were detected using a monkey polyclonal anti-SIV serum, and CIITA using the mouse 7-1H mAb. Proteins were revealed using the ECL system. Cells infected with FPwt were used as the negative controls.

Protein expression was investigated by WB after infection of replication-permissive CEFs and non-permissive Vero cells with the different recombinants. By infecting CEFs with FP*gp* or FP*gpCIITA*, the Gag protein was expressed although at different levels (Fig 2A). Similar results were shown for *env* gene expression after infection with FP*env* or FP*envCIITA* (Fig 2B). In contrast, in infected Vero cells, the expression of Gag and Env proteins was not affected by the presence of CIITA (Fig 2A and 2B). We therefore tested whether there was any difference in Vero cells when CIITA was driven by the H6 promoter rather than the SP promoter, with a high expression seen after infection with FP*CIITA*_H6_ (Fig 2C). In addition, when co-infection of Vero cells was performed with the FP*gp+*FP*CIITA*_H6_ or FP*env+*FP*CIITA*_H6_ recombinants, there were high levels of both *gp* and *env* expression. No specific bands were detected when CEFs and Vero cells were infected with FPwt (Fig 2A–2D).

### CIITA induces HLA-DR cell-surface expression only when the FP*CIITA* recombinant is driven by the H6 promoter

**Fig 3:**
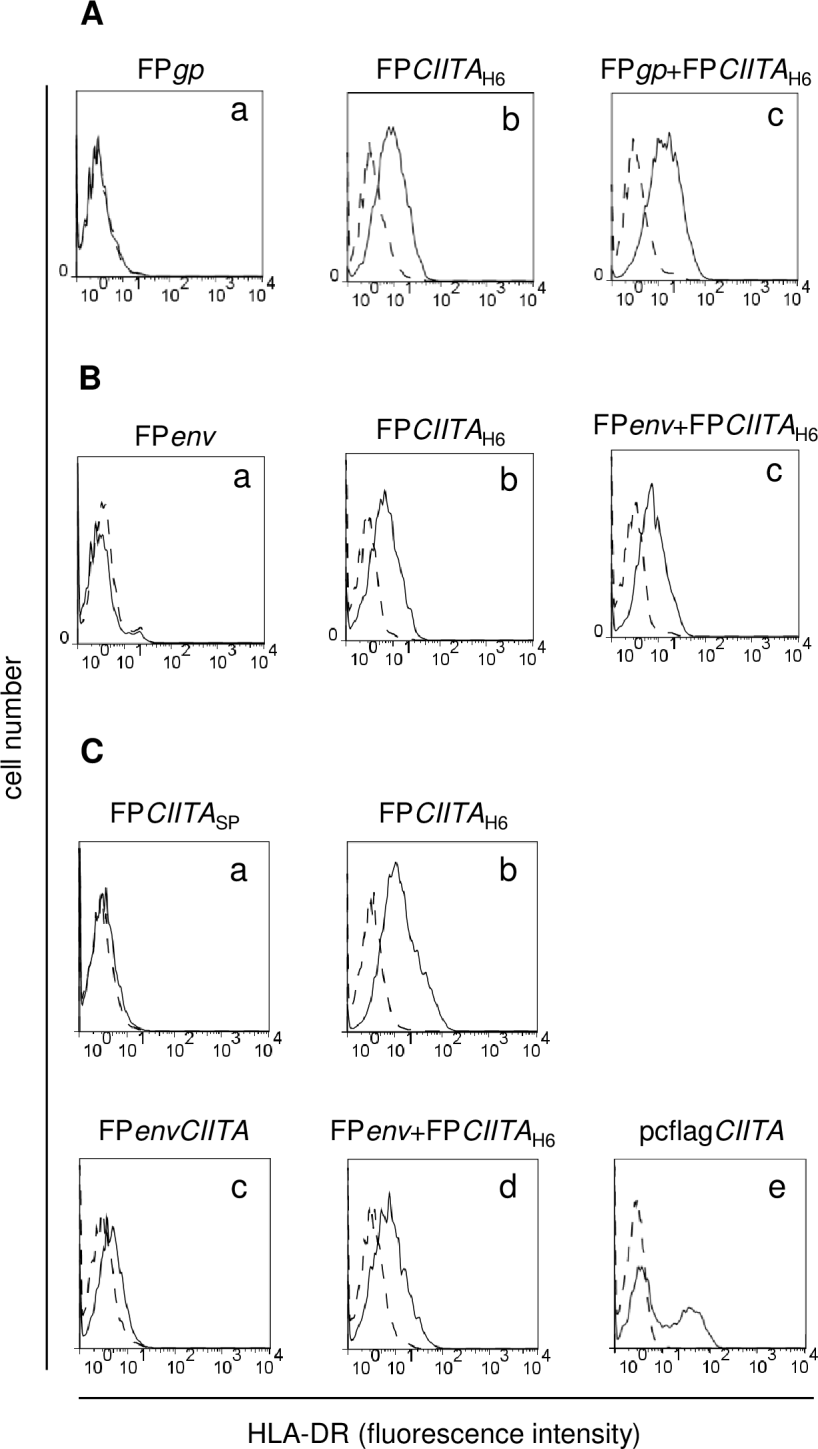
Analysis of CIITA-mediated HLA-DR trasactivation by FACS. After 293T cell infection, an increase in *HLA-DR* expression was mainly detected when the cells were infected with FP*CIITA*_H6_ (A, B, b *vs* a) or co-infected with the FP*gp*+FP*CIITA*_H6_ or FP*env*+FP*CIITA*_H6_ single recombinants (A, B, c). HLA-DR expression was enhanced when the CIITA transgene was driven by the H6 promoter (C, b *vs* a). *CIITA* better transactivates endogenous HLA-DR after co-infection with FP*env*+FP*CIITA*_H6_, rather than by the FP*envCIITA* double recombinant (C, d *vs* c). HLA-DR expression was observed in cells transiently transfected with pcflag*CIITA* (about 40% of the cells), as expected (C, e). The experiments were replicated twice with similar results. Results are expressed as number of cells *vs* the intensity of fluorescence in arbitrary units. Histograms represent fluorescence profiles of the cells, incubated with specific anti HLA-DR mAb (solid line) or with FITC-conjugated F(ab)2 anti mouse antibody (dashed line).

Expression of CIITA-driven HLA-DR was verified by immunofluorescence and flow cytometry in 293T cells infected with the different recombinants. In cells infected with FP*CIITA*_H6_ (Fig 3A and 3B, b) or co-infected with the FP*gp*+FP*CIITA*_H6_ or FP*env*+FP*CIITA*_H6_ single recombinants (Fig 3A, 3B and 3C), CIITA promoted HLA-DR expression 3 days p.i. As WB showed that the CIITA protein levels were low when expressed by FP*CIITA*_SP_, we investigated whether this might also affect the promotion of HLA-DR expression. Fig 3C shows that only the cells infected with the FP*CIITA*_H6_ recombinants express HLA-DR (Fig 3C, b *vs* a), which suggests that the amount of CIITA is crucial to induce HLA-DR transcription. This was confirmed in cells infected with the FP*envCIITA* double recombinant, in which CIITA expression was under the control of the SP promoter where HLA-DR expression was not observed (Fig 3C, c), whereas HLA-DR expression was induced after co-infection of FP*env*+FP*CIITA*_H6_ (Fig 3C, d). Similar data were obtained for FP recombinants expressing the *gp* gene (data not shown). No differences were observed between infection with 8+8 or 4+4 PFU/cell, and thus only infections with the former are shown here. As expected, 293T cells transfected with pcflag*CIITA* showed a CIITA-positive cell population that expressed HLA-DR on the cell surface (Fig 3C, e).

### CIITA does not affect the SIV Env and Gp subcellular distributions

**Fig 4:**
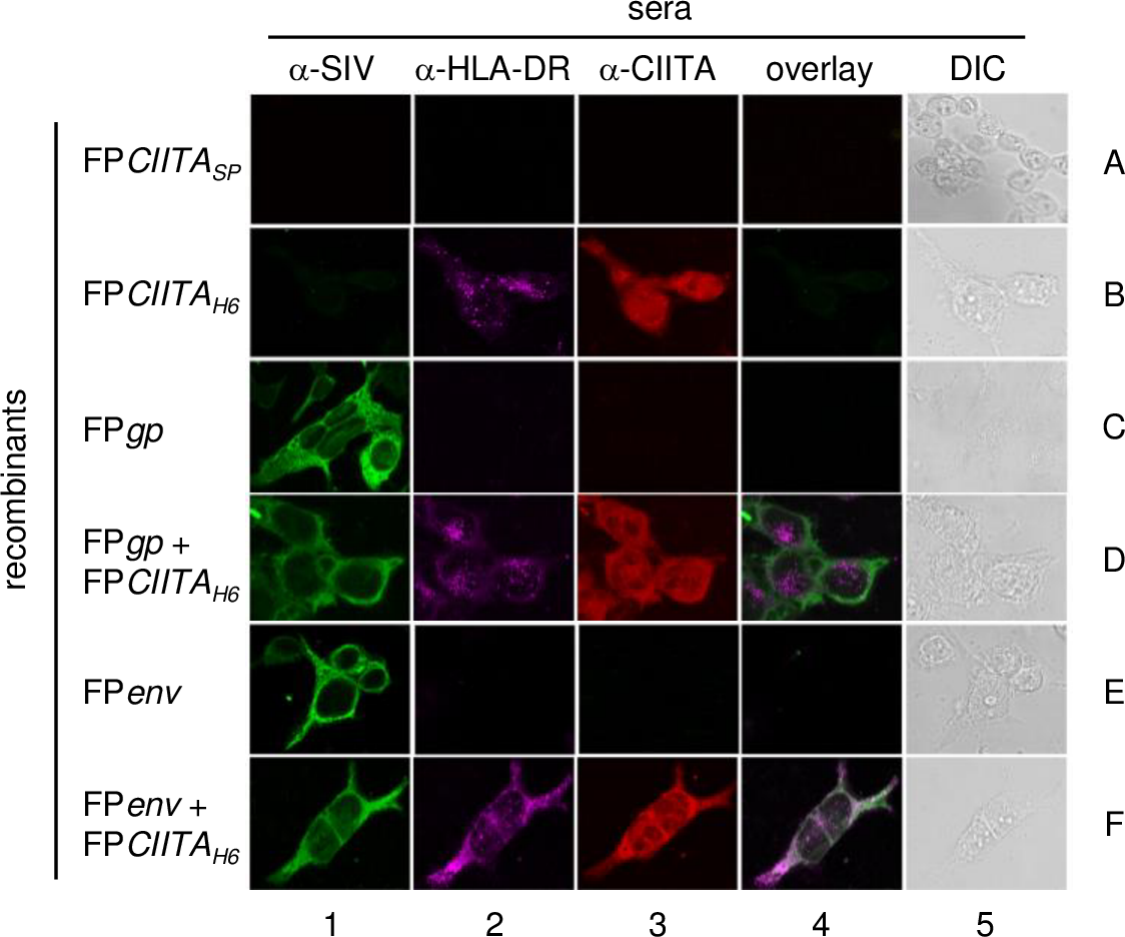
CIITA subcellular localisation using confocal microscopy. The 293T cells were infected with different FP recombinants and analysed using an anti-SIV polyclonal antibody, and anti-HLA-DR and anti-*CIITA* mAbs. Using anti-SIV serum, the Gp and Env proteins were equally well expressed by FP*gp* or FP*env* (C1, E1), as well as by FP*gp*+FP*CIITA*_H6_ (D1) or FP*env*+FP*CIITA*_H6_ (F1). After infection with FP*CIITA*_H6_, both HLA-DR and CIITA were expressed (B2, B3). Also, HLA-DR is expressed in cells co-infected with FP*gp*+FP*CIITA*_H6_ (D2) or FP*env*+FP*CIITA*_H6_ (F2). As expected, no Gp or Env proteins were present after infection with the FP*CIITA*_SP_ or FP*CIITA*_H6_ recombinants (A1, B1), and no HLA-DR or CIITA expression when the cells were infected with FP*gp* (C2, C3) or FP*env* (E2, E3). Differential interference contrast (DIC) microscopy of 293T cells is also shown (A5-F5).

To determine the subcellular localisations of the SIV Gp and Env, HLA-DR, and CIITA proteins, 293T cells were infected with different FP recombinants and analysed by immunofluorescence and confocal microscopy (Fig 4). These data showed that the HLA-DR and CIITA proteins were expressed when the infection was performed with the FP*CIITA*_H6_ recombinant, but not with the FP*CIITA*_SP_ recombinant (Fig 4, B2 *vs* A2, B3 *vs* A3, respectively). CIITA was expressed both in the cytoplasm and the nucleus (Fig 4, B3, D3, F3), which confirmed previous studies [53, 54], whereas HLA-DR showed punctate staining on the cell membrane (Fig 4, B2, D2, F2). The expression of the SIV Gp or Env proteins did not modify the localisation of both HLA-DR and CIITA (Fig 4, D2, D3, and F2, F3 *vs* B2, B3). Conversely, HLA-DR and CIITA were not expressed in cells that were infected with the FP*gp* (Fig 4, C2, C3) or FP*en*v (Fig 4, E2, E3) single recombinants, or with the FP*gpCIITA* or FP*envCIITA* double recombinants (data not shown). These data are in line with the HLA-DR expression on the cell surface of infected 293T cells revealed by FACS analysis (Fig 3). To determine whether CIITA can affect the subcellular localisation of the SIV proteins, the distributions of the Env and Gp proteins were analysed both in the absence and presence of *CIITA*. These data showed that both Gp and Env were localised in the cytoplasm of cells infected with the FP*gp* or FP*env* single recombinants (Fig 4, C1, E1, respectively). Co-infection using FP*CIITA*_H6_ + FP*gp* or + FP*env* did not affect the subcellular distributions of the SIV viral proteins (Fig 4, D1, F1). No Env or Gp proteins were present after infection with the FP*CIITA*_SP_ or FP*CIITA*_H6_ recombinants (Fig 4, A1, B1), as expected. Similar data were obtained using the T/SA cells (data not shown).

### Co-infection of T/SA cells with FP*env*+FP*CIITA*_H6_ does not increase the humoral immune response elicited in mice by T/SA cells infected by FP*env* alone

**Fig 5:**
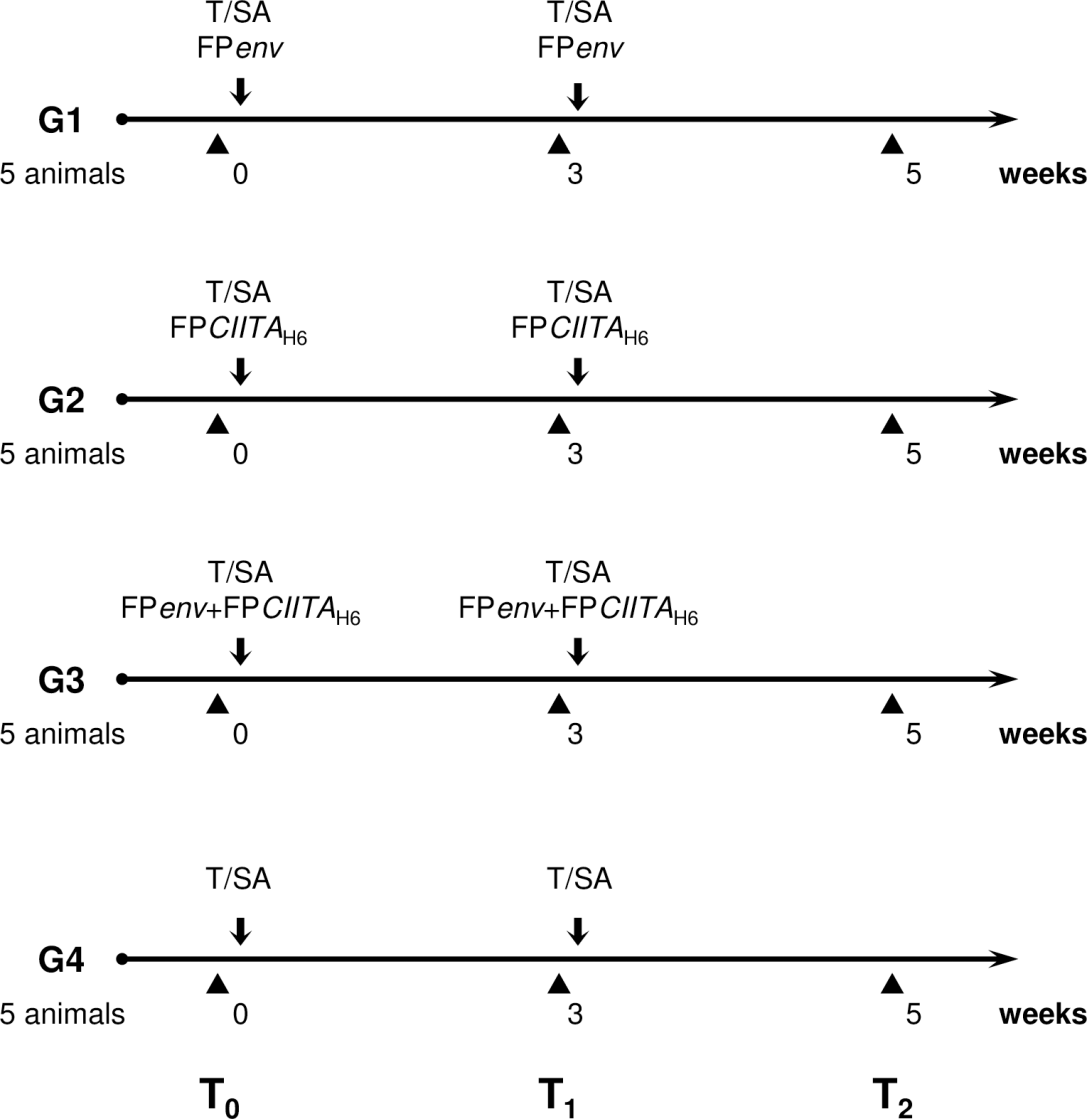
*In-vivo* experimental vaccination regimens. Five Balb/c mice per group were used to evaluate the humoral immune responses following their injection with T/SA cells infected or co-infected with FP single recombinants. The mice were immunised twice at a 3-week interval. Black triangles, times of mice bleeding; arrows, cell inoculations.

**Fig 6:**
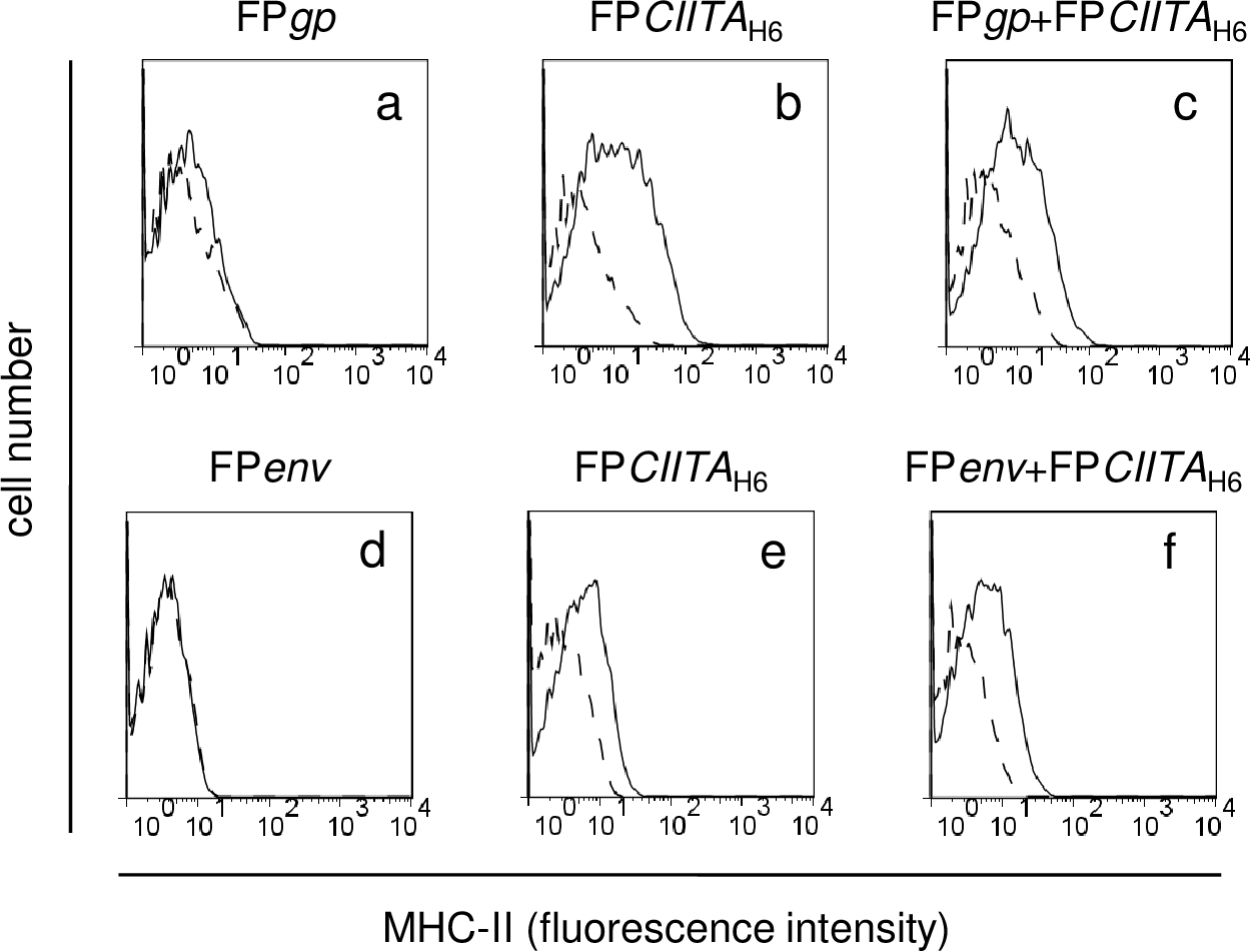
CIITA functionality in T/SA cells using FACS analysis. MHC-II expression was examined after infection of T/SA cells with the FP*gp*, FP*env*, FP*CIITA*_H6_, or FP*gp*+FP*CIITA*_H6_/ FP*env*+FP*CIITA*_H6_ recombinants. Similar data as those for 293T cells were obtained, which confirmed that MHC-II expression was higher when using FP*CIITA*_H6_ alone (b, e) or in combination with FP*gp* or FP*env* (c, f), rather than when the cells were infected with FP*gp* or FP*env* alone (a, d). Results are expressed as number of cells *vs* the intensity of fluorescence in arbitrary units. Histograms represent fluorescence profiles of the cells, incubated with specific anti MHC-II mAb (solid line) or with FITC-conjugated F(ab)2 anti mouse antibody (dashed line).

To determine the intensity of the immune responses against viral products when the antigens were expressed in cells carrying MHC-II, Balb/c mice were inoculated with infected syngeneic tumour T/SA cells, according to the scheme indicated in Fig 5. CIITA-driven mouse MHC-II expression in infected T/SA cells, investigated by FACS analyses was observed in cells infected with FP*CIITA*_H6_ alone (Fig 6, b, e) or in combination with FP*gp* (Fig 6, c) or FP*env* (Fig 6, f). This was not observed in cells infected with only FP*gp* (Fig 6, a) or FP*env* (Fig 6, d), confirming the results obtained with the 293T cells. Env expression was also confirmed (data not shown).

**Fig 7:**
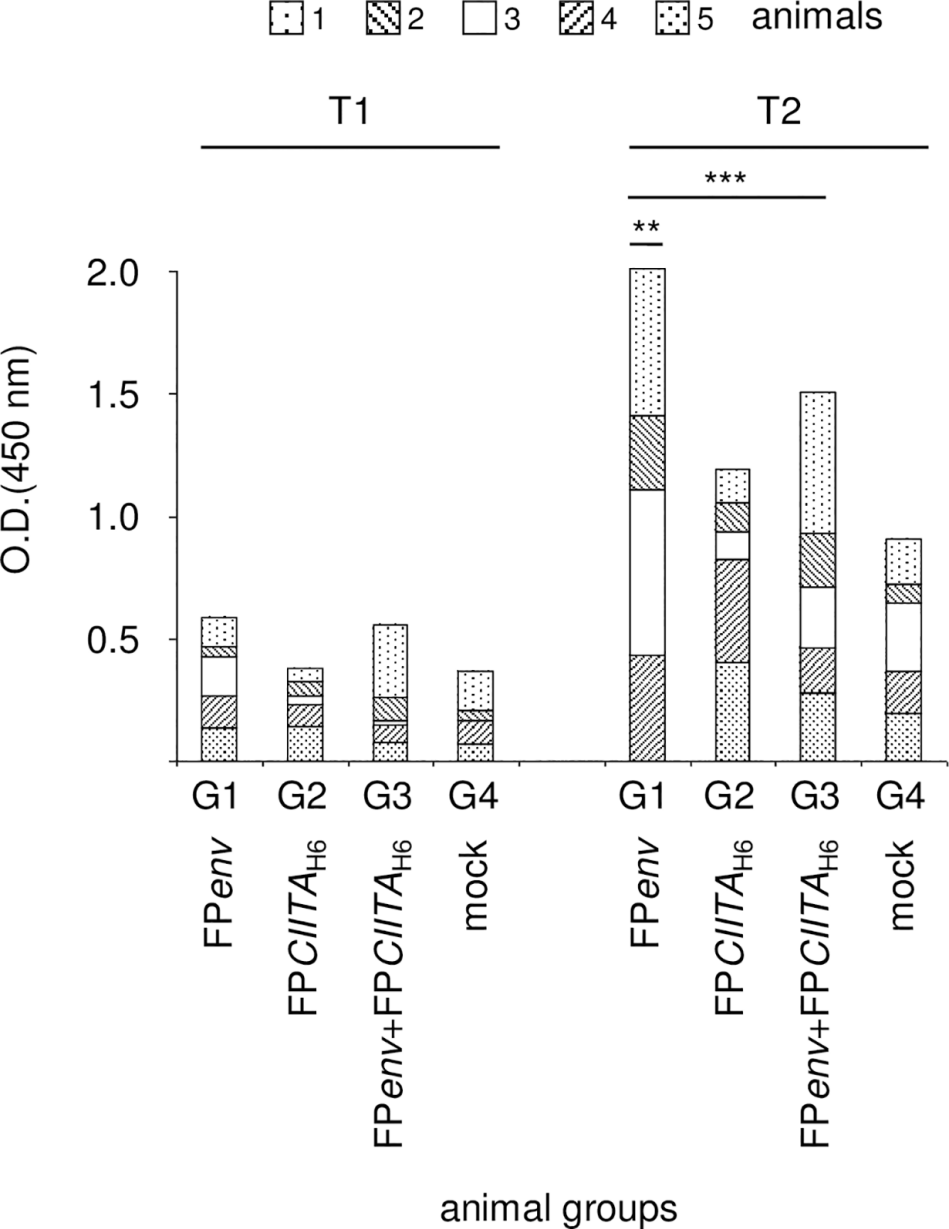
Analysis of the humoral response. The anti-Env humoral response was determined using ELISA, with lysed Vero cells previously infected with FP*env* as the plate-bound antigen. Serum was obtained from all of the mice at T0, T1, T2 and examined. After the second inoculation of T/SA cells, significant increases in the antibody titres were observed in G1, G2 and G3 (T2 *vs* T1; p <0.001). Also, a significant difference is shown when T/SA cells were infected only with FP*env* rather than after co-infection with FP*env*+FP*CIITA*_H6_ (T2, G1 *vs* G3; p <0.01). The OD_450_ values were expressed after subtracting the T0 values for each mouse. Statistical significances using one-way ANOVA parametric tests and Bonferroni analysis of variance are shown: p <0.01 (**), p <0.001 (***).

The blood of mice injected with infected T/SA cells was drawn to assess the Env-specific antibody response by ELISA. Although significant increases were found after the second inoculation (Fig 7, T2 *vs* T1; p <0.001), no differences among the groups of mice are shown after the first injection (Fig 7, T1). After the second inoculation, a significant difference is shown when T/SA cells were infected only with FP*env* (Fig 7, T2, G1 *vs* G3; p <0.01). The OD_450_ values were expressed after subtracting the values of the pre-immune serum (T0) for each mouse.

## Discussion

With the continued spread of acquired immunodeficiency syndrome, there is an urgent need for an effective preventive HIV vaccine, although the absence of clear immune correlates of protection makes this ultimate goal very difficult to be achieved. Although the present putative vaccines do not provide protection against HIV/SIV challenges by T-cell responses, CD8^+^ T cells have a major role in the control of viral replication [75–77]. Specific CD8^+^ T cells can maintain a stable viral load, attenuate acute viral replication in infected animals [75, 78], and favour the decline of viremia. Also, their experimental depletion has lead to SIV replication in infected macaques [79], and they are mainly responsible for viral clearance and protection against disease [80, 81], and might synergise with antibodies induced by vaccination. It has also been shown that cytolytic-T-lymphocyte-mediated immune responses precede the appearance of neutralising antibodies by many weeks [82], and are induced by recognition of virus-infected host cells.

It is generally assumed that a protective vaccine should elicit broadly neutralising antibodies that act at the serum and mucosal levels to prevent infection and viremia [83]. Indeed, passive transfer of broadly neutralising antibodies has blocked SHIV infection in non-human primates [84, 85] and delayed HIV-1 rebound after cessation of antiretroviral therapy in humans [86]. Studies in humanised mice and non-human primates have also shown protection against HIV-1 and SHIV infection by antibody delivery [87]. For this purpose, the use of the CIITA transactivator, which has a major role in triggering immune responses, might induce the expression of MHC-II molecules and improve antigen presentation to CD4^+^ Th cells.

It was generally evident that *gp* and *env* transgene expression by the FP*gp* or FP*env* single recombinants and by the FP*gpCIITA* or FP*envCIITA* double recombinants was lower than after co-infection with the FP*gp+*FP*CIITA*_H6_ or FP*env+*FP*CIITA*_H6_ single recombinants. As for CIITA, it was better expressed when the recombinant was used alone than after co-infection with the other SIV genes, independently from the type of promoter driving its expression. However, in spite of the initial decrease, *CIITA* expression increased over time when co-infection was performed, and enhanced the expression of both *gp* and *env* transgenes. If a lower expression level can be expected by the presence of two different transcripts in the same cell after infection with the double recombinant, this is in contrast with higher *gp* and *env* transgene expression during co-infection with single recombinants in association with FP*CIITA*_*H6*_. We can hypothesise that co-infection with two FP recombinants can benefit from the doubling of the RNA polymerases carried by the poxviral vectors, and thus increased transgene expression. Due to the presence of greater numbers of viral particles, co-infection might induce a more effective shutdown of the host cell proteins, to result in higher expression of the foreign viral genes.

Although some studies have indicated the efficiency of the SP synthetic promoter, real-time PCR data have also shown that the FP*CIITA*_SP_ single recombinant expresses *CIITA* less efficiently than its FP*CIITA*_H6_ counterpart. This might explain the lower expression of the *gp* and *env* transgenes by the double recombinants, where *CIITA* is driven by the synthetic SP promoter. For the same reason, *CIITA*_H6_ mRNA, which was expressed at higher levels by the single recombinant, decreased in the FP*gpCIITA* and FP*envCIITA* double recombinants, but regained the high levels at day 6 p.i. after co-infection with FP*gp* or FP*env*. These data are in agreement with the WB results performed on the Vero cells, where the *gp* and *env* proteins were very high after co-infection with the FP*gp+*FP*CIITA*_H6_ and FP*env+*FP*CIITA*_H6_ single recombinants when *CIITA* expression was driven by the H6 promoter. The better performance of the H6 promoter was also confirmed by FACS and confocal microscopy, as no HLA-DR expression was seen after infection with FP*CIITA*_SP_.

This was also demonstrated by the increased HLA-DR expression after co-infection of FP*CIITA*_H6_ with the FP*gp* or FP*env* recombinants, and the high CIITA expression by confocal microscopy using the same single recombinants, which was not observed after infection with the double recombinants where *CIITA* was under the SP promoter. These data show that FP*CIITA*_H6_ can express a functional CIITA that can modulate the induction of MHC-II HLA-DR molecules in human 293T cells that do not express CIITA endogenously. Confocal analysis also demonstrated the canonical dual subcellular localisation of CIITA and HLA-DR in the nucleus and in the cytoplasm, and that the SIV Gp and Env proteins did not affect their expression and localisation.

There are several strategies to enhance the immunogenicity of vaccines. One possibility is to increase the MHC-II-dependent antigen presentation, and thereby trigger the Th cell (CD4^+^) and cytolytic T-cell (CD8^+^) responses. Although it can be hypothesized that antigen-specific CD4^+^ cell activation may also generate new targets for HIV-1 infection, the risk of propagating productive infection is largely counterbalanced by CIITA expression, as a potent HIV-1 restriction factor, strongly inhibiting virus replication [59, 71].

In the present study, we also investigated the possibility to enhance the immune response against SIV structural proteins. Syngeneic T/SA tumour cells were used for this, which were infected with FP recombinants that expressed CIITA-induced MHC-II molecules, for better presentation of the relevant viral protein peptides to the CD4^+^ T cells. As FP*gp* constructs can release virus-like particles [18, 67, 88], and thus activate the immune presentation through MHC-I, only the FP*env* recombinants were considered to evaluate the contribution of CIITA to MHC-II activity in the antigen presentation and modulation of the humoral response.

## Conclusion

In the present study, we have demonstrated that: (i) *CIITA*_H6_ expressed by FP recombinants is functional; (ii) *CIITA* expression is higher when driven by the H6 promoter than by the synthetic SP promoter; (iii) SIV *gp* and *env* transgene expression is generally enhanced by co-expression of *CIITA* driven by the H6 promoter; (iv) CIITA expression is higher when carried by FP single recombinants than when co-expressed with FP*gp* or FP*en*v; (v) FP*CIITA*_H6_ transactivates human MHC-II and expresses HLA-DR molecules.

The failure of *CIITA* in enhancing the *env*-specific humoral immunity after co-infection with FP*env* was not so obvious and might be due to the presence of the second transgene. Also, the used ELISA was not characterised by high specificity and a further development of the assay is needed to draw conclusive data in future studies. As CIITA has been shown to potently enhance immunogenicity of tumour cells of different histotypes and distinct MHC genotypes by triggering tumour-specific CD4^+^ T cells [60], we can also hypothesise that the level of MHC-II molecules was too low to bind the viral *env* antigenic peptides, and might not reach the threshold for CD4^+^ T-cell stimulation. The T/SA cell system used might also have intrinsic limitations for the processing of sufficient amounts of the immunogenic *env* peptides to be presented by the MHC-II molecules. It will be also important to determine whether the recombinant SIV Env protein can access the endosomal compartment in which the protein processing, digestion and peptide loading on MHC-II molecules take place. Further *in-vivo* experiments are required to evaluate the complete CD4^+^ and CD8^+^ immune responses. The use of *prime/boost* immunisation regimens and different administration routes of the recombinants may enhance the immunogenicity of Env peptides presented by MHC-II and induce CD4^+^ T-cell stimulation.

## Ethics statement

The study was approved by the Institutional Ethics Committee (University of Milan and University of Varese).

## Consent for publication

All of the authors have read and approved the present version of the manuscript.

## Abbreviations

CEFs: chick embryo fibroblasts
ELISA: enzyme-linked immunosorbent assay
WB: Western blotting
FP: fowlpox
HRP: horseradish peroxidase
CS: calf serum
FCS: foetal calf serum
P/S: penicillin/streptomycin
PBS^-^: Ca^2+^-free and Mg^2+^-free phosphate-buffered saline
PFU: plaque-forming unit
MHC-II: class-II major histocompatibility complex
CIITA: class II transactivator
